# Structured evaluation of the effectiveness of an interactive tool for developing interpretation skills in mammography

**DOI:** 10.1186/bcr3283

**Published:** 2012-11-09

**Authors:** R Pietrosanu, H Gay, A Patel, L Blot, M Hartswood, R Proctor, P Taylor, L Wilkinson

**Affiliations:** 1 St Georges Hospital NHS Trust, London, UK; 2 The Royal Surrey County Hospital NHS Foundation Trust, Guildford, UK; 3 University of York, UK; 4 University of Edinburgh, UK; 5 University of Middlesex, UK; 6 University College London, UK

## Introduction

Lesion Zoo, a computer-based training tool, was developed to give trainee radiologists access to a wide range of possible mammographic appearances. Users describe lesions using terms from the BI-RADS lexicon and are given feedback on how their descriptions compare with those of by a panel of experts.

## Methods

A zoo is a collection of lesions presented to a trainee, who is invited to ascribe features to the lesions and provide a confidence estimate. Trainees receive feedback on their progress, through a detailed comparison of their assessment with that of a panel of experts, and from a learning curve showing how their confidence changes. During this evaluation, two sets of test cases (masses and calcifications) were offered initially as a baseline assessment, followed by training on masses or calcifications, followed by a second assessment after which training was swapped (the group that had initially trained on masses was trained on calcifications and *vice versa*), and then a third assessment was carried out, allowing a test of the impact of the training on performance.

## Results

Analysis of variance shows significant two-way interaction: participants receiving training on calcifications improve more on calcifications than on masses and participants receiving training on masses improve more on masses.

## Conclusion

Computer-based training tools provide a valuable addition to conventional training and allow trainees to get rapid access to experience with large numbers of cases. Well-designed tools that provide meaningful feedback on interpretation tasks are likely to be effective in improving performance.

